# Scaling of Individual Phosphorus Flux by Caterpillars of the Whitemarked Tussock Moth, *Orygia leucostigma*

**DOI:** 10.1673/031.009.4201

**Published:** 2009-06-18

**Authors:** T. D. Meehan, R. L. Lindroth

**Affiliations:** Department of Entomology, University of Wisconsin, Madison, Wl 53706

**Keywords:** allometry, body mass, compensatory feeding, consumer-driven nutrient recycling, ecological stoichiometry, environmental temperature, excretion, Lepidoptera, phosphorus, tussock moth

## Abstract

We conducted a laboratory study to evaluate the effects of body mass, environmental temperature, and food quality on phosphorus (P) efflux by caterpillars of the whitemarked tussock moth, *Orygia leucostigma*, J. E. Smith (Lepidoptera: Lymantriidae). We found that individual phosphorus efflux rate (*Q* the rate at which excreted and unassimilated P was egested in frass, mgP/day) was related to larval mass (*M,* mg dry) and environmental temperature (*T*,K) as *Q* = *e*^14.69^ *M*^1.00^e^-0.54/*kT*^, where *K* is Boltzmann's constant (8.62 × 10^-5^ eV/K, 1 eV = 1.60 × 10-19J). We also found that P efflux was not related to food phosphorous concentration, and suggest that this result was due to compensatory feeding by larvae eating low quality leaves. The P efflux model resulting from this analysis was simple and powerful. Thus, it appears that this type of model can be used to scale P flux from individual larvae to the population level and link species of insect herbivores to ecosystem processes.

## Introduction

Herbivorous insects play important roles in nutrient cycling in forest ecosystems. They consume canopy foliage, transform materials through digestive processes, and translocate portions of the canopy to the soil as frass (Mattson and Addy 1975; Hunter 2001; Schowalter 2006). The compounds in herbivore frass are then taken up by plants or microbes, stimulating microbial activity in the soil and affecting microbe-mediated ecosystem processes such as carbon and nutrient mineralization (Lovett and Ruesink 1995; Reynolds and Hunter 2001; Christenson et al. 2002; Frost and Hunter 2004).

Previous work on herbivore-mediated nutrient fluxes from the canopy to the soil has focused mainly on the movement of nitrogen (Swank et al. 1981 ; Lovett et al. 2002; Townsend et al. 2004). Relatively few studies have considered phosphorus flux by insect herbivores (Fogal and Slansky 1985; Hollinger 1986), despite the fact that phosphorus has been shown to limit primary production in both aquatic and terrestrial ecosystems (Elser et al. 2000; Elser et al. 2007).

A common approach to studying elemental fluxes via herbivore frass is to collect frass in baskets placed on the forest floor, weigh it, determine its elemental concentration, and calculate the total flux from the canopy to the soil (e.g., Hunter et al. 2003). This approach provides a flux estimate for the community as a whole, but is not useful for estimating the contribution of particular species. Alternatively, it may be possible to compute specieslevel estimates of elemental flux by combining predictions of individual fluxes with information on the characteristics of populations. This approach would facilitate linking the activities of a particular species to fluxes of elements in forested ecosystems.

In this study we use principles from animal physiology to develop a simple model of phosphorus efflux via frass that can be used to predict deposition by individuals over a range of body sizes, body temperatures, and food qualities. The model is evaluated using data on phosphorus efflux by caterpillars of the whitemarked tussock moth, *Orygia leucostigma*, J. E. Smith (Lepidoptera: Lymantriidae).

## Materials and Methods

### Model

Individual phosphorus efflux rate (*Q* mg P/day in frass) was modeled as a function of larval body mass (*M*, mg dry mass), environmental temperature (*T,* K), and leaf phosphorus concentration (*P_i_* % dry mass). The rates of several physiological processes, including elemental fluxes, frequently are related to body mass as a power function with a scaling exponent, *b*, between 0.65 and 0.85 (Peters 1983; Calder 1984; Schmidt-Nielsen 1984; Brown 1995; Brown et al. 2004). Thus, phosphorus efflux was modeled as *Q* α *M^b^* and *b* was expected to fall near 0.75.

Physiological rates also are known to increase exponentially with body temperature (Crozier 1924; Cossins and Bowler 1987; Gillooly et al. 2001; Hochachka and Somero 2002). Therefore, an exponential temperature term was added to the phosphorus efflux model so that *Q* α *M*^b^e^-*E/kT*^ Gillooly et al. 2001). This temperature term is referred to as the Arrhenius or Boltzmann factor (Arrhenius 1915; Gillooly et al. 2001). Although its roots are in physical chemistry (for describing the temperature dependence of simple chemical reactions), it has also been shown to be useful for describing the temperature dependence of many whole-organism physiological rates (Crozier 1924; Gillooly et al. 2001; Gillooly et al. 2002; Gillooly et al. 2005b). Here, *k* represents Boltzmann's constant (8.62 … 10^-5^ eV/K, 1 eV = 1.60 × 10^-19^J) and *E* reflects the average activation energy of the biochemical reactions involved in aerobic respiration (approximately 0.6 to 0.7 eV) (Gillooly et al. 2006).

Individual phosphorus efflux was expected to be a function of the phosphorus concentration of leaves for two reasons. First, this relationship would be expected if a fraction of the ingested phosphorus was never absorbed as food passed through the gut. Second, a surplus of phosphorus in food should lead to a decrease in phosphorus absorption or an increase in phosphorus excretion (Sterner et al. 1992; Elser and Urabe 1999; Sterner and Elser 2002; Woods et al. 2002). Together, these factors should result in a positive relationship between individual phosphorus flux and leaf phosphorus concentration that can be represented as *Q α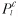.* A power function relationship with scaling exponent, *c*, is used because it is a simple and flexible function that can accommodate the potentially nonlinear relationship between individual flux and leaf phosphorus. Combining this relationship with those above gives a simple equation for predicting phosphorus efflux by an individual insect herbivore, *Q = a* *M^b^e^-E/kT^
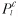
*, where a is a normalization constant with units mg P day^-1^ mg larva^-b^.

### Evaluation

To evaluate the individual phosphorus efflux model, a dataset was assembled that included information on larval mass, leaf phosphorus concentration, frass phosphorus concentration (*P_f_,* % dry mass), dry-matter egestion rate (*E,* mg dry/day), and individual phosphorus efflux rate (the product of frass phosphorus concentration and egestion rate) for larval *O. leucostigma. Orygia leucostigma* was used in this study because it is a widely distributed native herbivore, and because it has similar feeding behavior, gut chemistry, and frass chemistry (Frost and Hunter 2007) as other economically important species such as the gypsy moth, *Lymantna dispar.*

*Orygia leucostigma* larvae were raised from eggs purchased from the Canadian Forest Service (Sault St. Marie, Ontario, Canada). Egg masses were divided into two groups and put into two rearing dishes in a growth chamber set to 22° C and a 14:10 hr L:D cycle. Eggs hatched after two weeks of incubation, whereupon half of the larvae were fed high-nutrient leaves, while the other half were fed low-nutrient leaves. Leaves came from 10 potted aspen trees, *Populus tremuloides* Michx. (Malpighiales: Salicaceae), propagated from a single aspen clone (Waushara County, Wisconsin, USA). Trees were grown in 40 L pots, in a soil medium consisting of 70% sand and 30% silt-loam soil. Five of the 10 trees (high-nutrient trees) were given 4.5 g/L soil of slow-release fertilizer (18:6:12, N:P:K, without micronutrients) in May 2004 and 2007. Low nutrient trees were not given fertilizer. Trees were in their fourth growing season during summer 2006, when this study was conducted.

Phosphorus efflux data were collected during 24-hr feeding trials, which were conducted at various body masses over the course of larval development. In total, 25 trials were conducted using 129 larvae. The number of animals was greater than the number of trials because, in 15 cases, 2 to 11 similarly-sized individuals were placed into one vial for a feeding trial in order to collect enough frass for subsequent chemical analyses. For each trial, *O. leucostigma* larvae were weighed and a pre-trial wet mass was calculated as the total mass of larvae divided by the number of larvae. Then larvae were placed into a 20-ml polyethylene vial with a moist piece of tissue paper and 200 to 300 mg of high- or low-nutrient aspen leaves. Vials were then placed into environmental chambers set at 15 or 30° C and a 14:10 hr L:D cycle. After 24 hours, the vials were removed from chambers and placed directly into a freezer.

Samples were then freeze-dried and larvae and frass were weighed with a microbalance. Post-trial dry mass was calculated for each trial as the total dry mass of all larvae divided by the number of larvae in the vial. A pre-trial dry mass was estimated using pre-trial wet mass and the function: larval dry mass = 0.17 × wet mass^0.98^ (R^2^ = 0.99, n = 25). A midpoint dry mass was then calculated as the average of pre- and post-trial dry masses. A final larval mass for each trial was calculated by correcting midpoint average dry mass for gut contents using the equation: gut-content-free dry mass = 0.78 × midpoint dry mass^1.01^ (R^2^ > 0.99, n = 18). The gut content correction function was produced for the larvae in this study following the fasting method of Bowers et al. (1991). Drymatter egestion rate for each trial was calculated as the total dry mass of frass divided by the number of animals in a vial.

Dried leaf and frass samples were homogenized using a mortar and pestle, and phosphorus concentrations were quantified using the following modified version of the Murphy-Riley method (Murphy and Riley 1962; Olsen and Sommers 1982; Avila-Segura et al. 2004). First, samples of 3 mg of leaves, or 4 mg of frass, were combusted in 100 mm × 13 mm diameter glass culturing vials in a muffle furnace for 3 hours at 500° C. After cooling, 4 ml (for leaves) or 2 ml (for frass) of 1 M H_2_SO_4_ were added to ashes in vials to dissolve phosphorus in samples. After approximately 3 hr at room temperature, vials were centrifuged at 3500 rpm for 8 min. Next, a 100-µl sample of supernatant was combined with 100-µl of 2 M NaOH and 50 µl of freshly made Murphy-Riley reagent (Reagent 2, Olsen and Sommers, 1982) in the well of a 96-well microplate. On each plate, we included 2 replicates from each sample vial and 2 replicates from each of 5 levels of P standards (0, 0.5, 1, 2.5, 5 mg P/L, made from K_2_PO_4_ dissolved in 1 M H_2_SO_4_). Plates were then allowed to develop for 30 min at room temperature and were read at 850 nm using a 96-well-plate-compatible spectrophotometer. Repeated analysis of an internal leaf standard yielded an analytical coefficient of variation of 2.08 %.

As noted above, individual phosphorus efflux was expected to relate to larval mass, temperature, and leaf P concentration as *Q* = *a M^b^e ^-E/kT^* 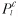 where *b* was expected to be near 0.75, *E* was expected to fall near 0.65 eV, and *c* was expected to be a positive number. We linearized this equation by taking natural logarithms of both sides and evaluated expectations by fitting the equation to natural log-transformed data using multiple linear regression. We used partial F-tests to evaluate the contribution of each term to the model, and calculated 95% confidence intervals to assess the uncertainty around estimated model coefficients.

## Results and Discussion

The phosphorus concentrations of the aspen leaves included in this study ranged from 0.11 to 0.20%, and averaged 0.15%. Phosphorus concentrations of tree foliage generally fall between 0.05 and 0.25% (Reich and Oleksyn 2004; Kerkhoff et al. 2005), so the leaves included in this study had phosphorus concentrations that were representative of the natural range of variation. The phosphorus concentrations of larval frass included in this study ranged from 0.03 to 0.13 % and averaged 0.07%. Rates of individual phosphorus efflux observed during the study ranged from 0.00032 to 0.076 mg P/day, while body masses and environmental temperatures ranged from 0.34–41.48 mg dry and 15–30° C, respectively.

**Figure 1.**
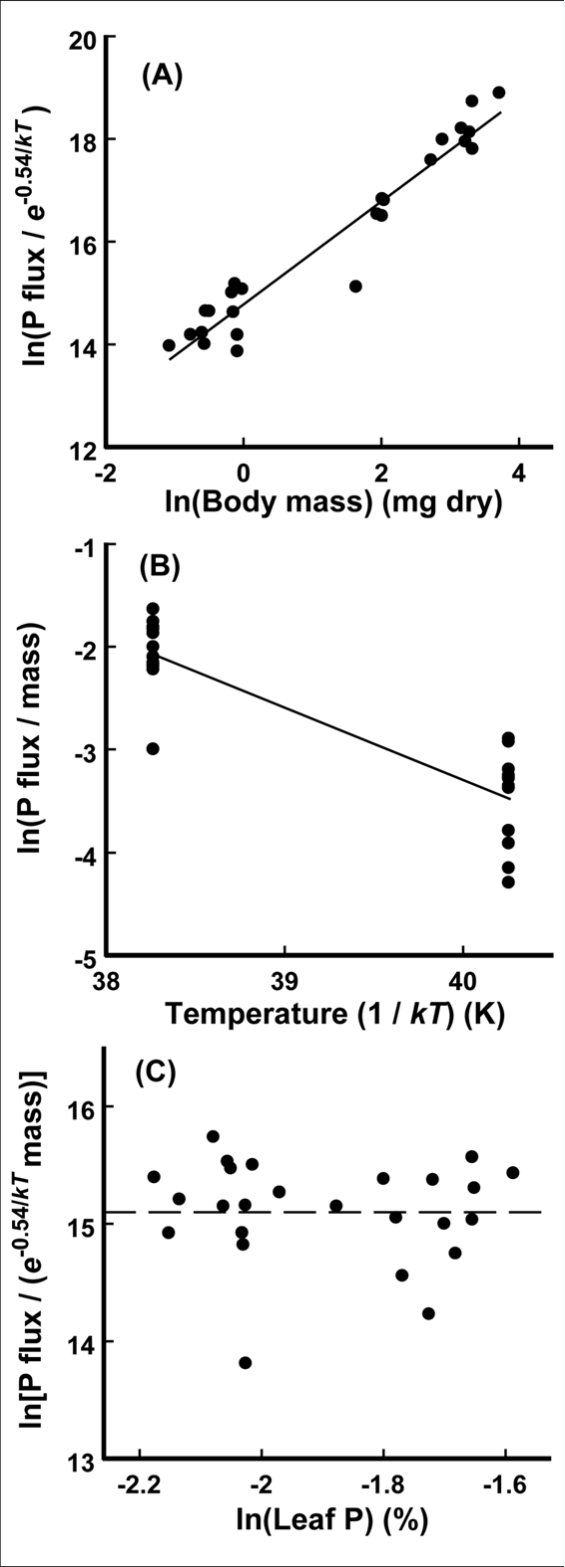
(A) Relationship between the natural log of larval body mass (mg dry) and the natural log of larval phosphorus flux (mg P/day) after accounting for the effect of environmental temperature (*T*, in K; k: is Boltzmann's constant, 8.62 × 10^-5^ eV/K, 1 eV = 1.60 × 10^-19^ J). (B) Relationship between the inverse of temperature multiplied by Boltzmann's constant and the natural log of larval phosphorus flux after accounting for the effect of larval body mass. (C) Relationship between the natural log of leaf phosphorus concentration (% dry mass) and the natural log of larval phosphorus flux after accounting for the effects of larval body mass and temperature.

Individual phosphorus efflux rate was found to be related to larval mass and environmental temperature as *Q* = e^14.69^*M*^1.00^e^-0.54*/kT*^ (R^2^ = 0.94, Figure 1). The 95% confidence intervals for the mass (F_1,22_ = 324.40, P < 0.001) and temperature (F_1,22_ = 34.87, P < 0.001) coefficients were 0.88 to 1.11 and -0.73 to -0.35, respectively. Phosphorus flux was related to leaf phosphorus concentration as *Q* ∝ *P_1_* ^-0.17^ , but the 95% confidence interval for the scaling exponent (-1.30 to 0.95) and partial F-test (F_1,21_ = 0.75, P = 0.75) indicated that leaf phosphorus was not a significant predictor of larval phosphorus flux (Figure 1).

The temperature coefficient of 0.54 eV for individual phosphorus efflux was not significantly different from the range of 0.6 to 0.7 eV that is expected for metabolic rate (Gillooly et al. 2001; Gillooly et al. 2006). This coefficient can be translated into a *Q*_10_ value (factorial rate increase with a 10°C increase in temperature) using the equation *Q*_10_ = 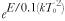 where *T_0_* is the median of the temperature range (K) over which temperature dependence was measured (Gillooly et al. 2001; Vasseur and McCann 2005). When this was done, a *Q1_0_* value of 2.05 was obtained for individual phosphorus flux, which falls within the range of 2–3 that is typically reported for metabolic rate (Withers 1992; Hill et al. 2004).

Relatively little has been published on the temperature dependence of individual elemental flux rates, *per se*, and most of the available information is on the temperature dependence of nutrient excretion by aquatic organisms. Wen and Peters (1994) conducted an analysis of the determinants of phosphorus excretion by zooplankton using data compiled from the literature. They found an average *Q*_10_ value of 1.6 for phosphorus excretion rates, and reported that other reviews (Ejsmont-Karabin 1984) have given *Q*_10_ values as high as 2.6. Whether these results are comparable to ours is not clear, however, because studies of aquatic herbivores generally have not quantified phosphorus fluxed via feces.

The mass-scaling exponent of 1.00 for individual phosphorus efflux rate was steeper than the range of 0.65 to 0.85 that is commonly reported for the metabolic rate of animals, in general (Hemmingsen 1960; Peters 1983; Gillooly et al. 2001; Savage et al. 2004; Glazier 2005; White et al. 2007), and for larval lepidopterans, in particular (Smith 1972). When possible causes for the steep mass-scaling coefficient were explored, it was found that the phosphorus concentration of frass was related to body mass as *P_f_* ∝ *M^0.13^*, i.e., frass phosphorus concentration increased with larval mass. In addition, total body phosphorus concentration of larvae, *Pb*,was related to body mass as *P_b_*, ∝ *M^-0.04^* , i.e., large larvae had relatively low body phosphorus concentrations (Woods et al. 2004; Bertram et al. 2008). Together, these results lead us to hypothesize that the mass scaling of phosphorus flux was steeper than expected because (1) larger larvae have lower concentrations of, and requirements for, phosphorus (Sterner and Elser 2002), (2) lower phosphorus requirements allow for lower phosphorus absorption or higher excretion, leading to (3) relatively high phosphorus concentrations of frass and overall phosphorus flux rates for relatively large larvae (Gillooly et al. 2005a). Further studies are necessary to evaluate this possibility.

We are not aware of previous studies on the mass-dependence of phosphorus flux by terrestrial herbivores, although several studies have addressed the allometry of phosphorus excretion by aquatic herbivores. Those studies have reported mass-scaling exponents between 0.62 and 0.69 for zooplankton Johannes 1964; Peters and Rigler 1973; Wen and Peters 1994) and between 0.78 and 0.89 for fish (Schaus et al. 1997; Vanni et al. 2002). Again, the degree to which these results are comparable to ours is not known because studies of aquatic herbivores generally have not quantified phosphorus fluxed via feces.

We were somewhat surprised to see that individual phosphorus efflux rate was unrelated to leaf phosphorus concentration. This likely occurred because a decrease in leaf phosphorus was accompanied by both a decrease in frass phosphorus concentration (*P_f_* ∝ 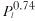) and an increase in larval ingestion and egestion rates (*E* ∝ 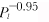 ), with the net result being an approximate independence of individual phosphorus flux and leaf phosphorus concentration(*Q* ∝ *E P_f_* ∝ 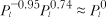)

**Figure 2.**
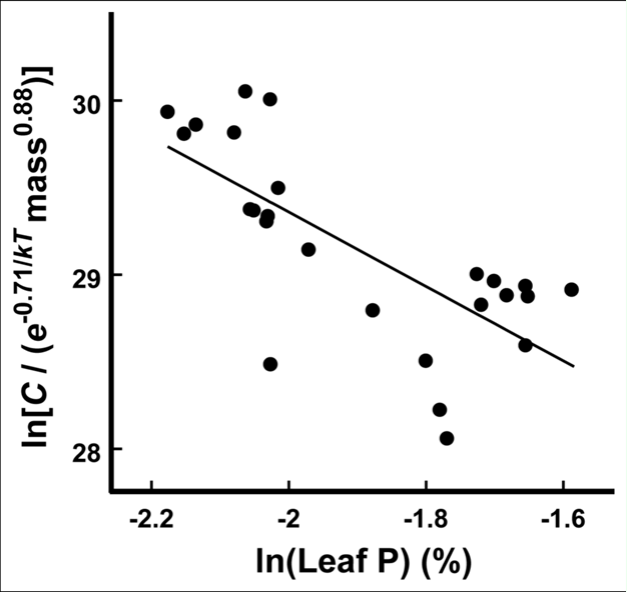
Relationship between the natural log of leaf phosphorus concentration (% dry mass) and the natural log of leaf consumption (*C,* mg dry/day) after accounting for the effects of larval body mass (mg dry) and environmental temperature (T, in K; *k* is Boltzmann's constant, 8.62 × 10^-5^ eV/K, 1 eV = 1.60 × 10^-19^ J). Consumption was calculated as dry mass of leaves before trial minus dry mass after trial divided by the number of individuals in trial. The negative relationship (F_1,21_ = 19.21, P < 0.001) suggests compensatory feeding by larvae in response to nutrient content of leaves.

The increase in ingestion and egestion rates by larvae given low-nutrient food is an example of compensatory feeding (Figure 2). Compensatory feeding in response to low food nitrogen concentrations has been observed in both terrestrial (Slansky and Feeny 1977; Raubenheimer 1992; Lindroth et al. 1997; Kingsolver and Woods 1998; Lavoie and Oberhauser 2004) and aquatic (Cruz-Rivera and Hay 2000; Fink and Von Elert 2006) organisms. Compensatory feeding in response to low food phosphorus concentrations has been explored for a variety of organisms, including lepidopterans (Woods et al. 2002; Perkins et al. 2004), though it has been clearly demonstrated only for snails (Fink and Von Elert 2006). In our study, the phosphorus and nitrogen concentrations of leaves were strongly correlated (r = 0.98), because high leaf phosphorus concentrations were achieved by adding a combined N, P, and K fertilizer to potted aspen trees. Consequently, it is quite possible that the compensatory feeding that was observed was a behavioral response to shortages in leaf nitrogen. Nonetheless, nitrogen and phosphorus concentrations of foliage tend to be correlated in the wild (r = 0.84, Garten 1976). Thus, our results may reflect the relationship between food quality and phosphorus flux in natural settings, and suggest that compensatory feeding should be considered in more detail in future theory on consumer-driven nutrient recycling (Elser and Urabe 1999; Sterner and Elser 2002).

The model for individual phosphorus efflux derived and evaluated here was relatively simple and powerful, in that it incorporated two easily measured predictor variables and explained 94% of the variation in individual phosphorus efflux rates. Thus, simple individual flux models such as these may provide a practical means for scaling nutrient deposition rates from individual larvae to population levels. With further work, it may be possible to (1) generalize the model for other species, (2) scale from individual elemental flux rates to whole communities of lepidopteran larvae, and (3) use these estimates to better understand the role of these organisms in the functioning of forest ecosystems.
